# Two Decades and Counting Since the Abuja Summit: Where Do We Stand in the Fight Against HIV/AIDS-Related Maternal Mortality?

**DOI:** 10.1177/26884844251386289

**Published:** 2025-10-08

**Authors:** Richmond Nketia, Austin Gideon Adobasom-Anane, Sandra Mensah, Faustina Ameyaa Marfo, Hannatu Favour Kachiro, Rostand Dimitri Messanga Bessala, Naomi Adotei, Abubakr Ahmed Farhan, Charles Limula, Ebenezer Gyamfi, Gideon Asamoah, Daniel Atta-Nyarko

**Affiliations:** ^1^ Department of Research and Data Analytics, Sub-Saharan Africa Research for Sustainable Development, Berekum, Ghana.; ^2^ Department of Global Health Policy and Development, Sub-Saharan Africa Research for Sustainable Development, Berekum, Ghana.; ^3^ Global Health and Infectious Diseases Research Group, Kumasi Centre for Collaborative Research in Tropical Medicine, Kwame Nkrumah University of Science and Technology, Kumasi, Ghana.; ^4^ Department of Community Health, College of Health - Yamfo, Yamfo, Ghana.; ^5^ Department of Health Administration, Brooks College of Health, University of North Florida, Jacksonville, Florida, USA.; ^6^ Department of Health Sciences, Faculty of Sport, Technology and Health Sciences, St. Mary’s University, Twickenham, United Kingdom.; ^7^ Department of Obstetrics and Gynaecology, Ahmadu Bello University Teaching Hospital, Zaria, Nigeria.; ^8^ Department of Internal Medicine, University of Yaounde I, Yaounde, Cameroon.; ^9^ Department of Paediatrics, St. Luke Hospital, Kasei, Ghana.; ^10^ Department of Clinical Care, Savannah Regional Health Directorate, Damango, Ghana.; ^11^ Skerne Medical Group (NHS), Harbinson House, Front St, Sedgefield, United Kingdom.; ^12^ Department of Medicine, Methodist Hospital, Wenchi, Ghana.; ^13^ Institute of Clinical Trials and Methodology, University College London, London, United Kingdom.; ^14^ Allied Health Professions Council (AHPC), Ministry of Health, Accra, Ghana.

**Keywords:** Africa, Abuja Summit, HIV/AIDS, Maternal Mortality

## Abstract

**Background:**

It is more than two decades since the Organisation of African Unity’s historic Abuja Summit, yet the fight against human immunodeficiency virus/acquired immunodeficiency syndrome (HIV/AIDS)-related maternal deaths in the region remains as critical as ever. This study examined four key dimensions to inform future actions: cross-national comparison of current HIV/AIDS-related maternal deaths, temporal trends in mortalities (for pre- and post-Abuja periods), variations across age groups and birth cohorts, and potential policy levers to drive progress.

**Methods:**

Poisson regression-based age-period-cohort models were fitted to the Global Burden of Disease Study 2021 (GBD 2021) data (1982–2021; *N* = 47,750 HIV/AIDS-aggravated maternal mortalities) to estimate age, calendar period, and birth cohort effects on mortalities for each of the 54 African countries.

**Results:**

Notable variations in HIV/AIDS‐aggravated maternal deaths were observed across Africa. In 2021, 16 of the 54 countries had age-standardized mortality rates near zero, while 38 countries reported rates ranging from 0.07 to 0.95 per 100,000 women. Mortality rates rose sharply from the early 1980s, peaked during the 1990s and early 2000s, and generally declined after the Abuja Summit; however, the trajectories varied considerably across the continent. Mortality also increased with age, with substantial heterogeneity in country-level patterns. In more than two-thirds of countries, cohort comparisons relative to a 1967 baseline showed marked increases in mortalities in recent birth cohorts.

**Conclusions:**

Given the marked variations in HIV/AIDS‐aggravated maternal mortality among African populations, this study advocates for context-specific, life-course strategies to drive progress toward universal health coverage and improved maternal health across Africa.

## Introduction

In April 2001, the Heads of State and Government of the Organisation of African Unity (OAU) met in Nigeria’s capital city, Abuja, at a special summit dedicated to addressing the burden and threats caused by human immunodeficiency virus and acquired immunodeficiency syndrome (HIV/AIDS), tuberculosis (TB), and other related infectious diseases.^1^ Beyond the “politics,” this historic meeting, enshrined in the “Abuja Declaration,” was also a call for African regional health transformation. At that moment, hope was high; the OAU member states declared their commitment to a resource-intensive, coordinated effort aimed at curbing the HIV/AIDS epidemic and, most importantly, safeguarding the health of the African population, particularly mothers who are most vulnerable to the disease and its long-term physical, psychological, and socioeconomic repercussions.^2,3^ Specifically, they pledged to allocate at least 15% of their annual budget to the national health sector,^4^ and urged donor countries to fulfil the yet to be met target of 0.7% of their gross national product as official development assistance (ODA) to developing countries.^5^

However, in the postsummit period, HIV/AIDS treatment, maternal care, and public health systems have evolved considerably across Africa, reflecting an era of new priorities, challenges, and innovations. Amid these evolutions, progress seems unappreciable, in both the OAU governments’ commitments to meet health targets and the ODA received from donor countries. A World Health Organization (WHO) report indicates that although 26 countries have increased the proportion of total government expenditures allocated to health, only Tanzania has achieved the Abuja Declaration target of “at least 15%.” Meanwhile, 11 countries reduced their relative contributions of government health spending between 2001 and 2011.^5^ Consequently, while the rapid scale-up of antiretroviral treatment (ART) and targeted interventions, especially those aimed at preventing mother-to-child transmission, have ushered in a new era of clinical breakthroughs and increased survival rates globally,^6–9^ the persistent gap between policy intentions and real-world implementation continues to expose many African women to a health care system that, despite its advancements, still struggles with resource constraints, socioeconomic disparities, and systemic inequalities.^10–14^ This raises concerns about the sustainability of current intervention models and the ability of health systems to deliver equitable, high-quality care to all women across the continent.^5^

To guide future policy directions and interventions, population studies must not only quantify the overall burden of HIV/AIDS but also examine how distinct temporal factors such as age, calendar period, and birth cohort shape these outcomes. As the first step in this process, it is crucial to precisely characterize the country-level epidemiological profile of HIV/AIDS-related maternal deaths by disaggregating the data according to age-specific rates, key historical milestones, and birth cohort trends. These detailed stratifications can help identify the specific periods and demographic groups where HIV/AIDS has exerted the strongest adverse effects, thereby enabling a more targeted response and efficient use of limited resources. While many epidemiological studies exist,^15–18^ those aiming to address these concerns have often combined HIV/AIDS data without breaking down into maternal mortality trends, an approach that obscures the unique vulnerabilities of different age groups and birth cohorts and hinders targeted interventions.

In this study, we examined not only the measurable changes in HIV/AIDS-exacerbated maternal death, but also the deeper, often less visible, inequalities across demographic groups and populations in Africa. In the sections that follow, we provide a detailed description of our study methodology, present the key findings from our age-period-cohort (APC) modeling, and critically interpret how HIV/AIDS has shaped maternal mortality across different periods and demographic segments in Africa. We then discuss the policy implications of these trends and offer targeted recommendations for ongoing public health interventions designed to address HIV/AIDS-related maternal deaths in Africa.

## Materials and Methods

### Conceptual framework

It is well established that cumulative exposures over the life course shape individual health outcomes by influencing two key dimensions, as demonstrated in life course theory and the cumulative advantage/disadvantage model: (1) intrinsic capacity (individuals’ underlying biological reserves that determine their potential to maintain health) and (2) functional ability (individuals’ capacity to effectively utilize these biological reserves within a supportive socioenvironmental context).^19^ Aligning with this background literature, our conceptual framework supports the model that exposures from birth through adulthood collectively contribute to later maternal health outcomes, including HIV/AIDS-related deaths.

At the macro-level (structural level), exposures such as the Abuja Declaration serve as key examples of policy efforts aimed at addressing the HIV/AIDS burden and maternal health issues.^1^ Although there is limited evidence regarding causality, the literature seems to suggest a beneficial association between such landmark policy directions and subsequent improvement in public health strategies for HIV/AIDS and maternal health through increased government health spending, expansion of ART access, and the implementation of comprehensive prevention of mother-to-child transmission (PMTCT) programs.^5,20,21^ In this context, these policies could create an enabling environment that identifies and mitigates early adverse exposures (thereby potentially enhancing intrinsic capacity) and nurtures conditions under which health-promoting behaviors can flourish.^22,23^

At the micro-level, individual behaviors and practices such as HIV self-testing, consistent adherence to safe sexual practices, and proactive utilization of available health care services play a crucial role in HIV/AIDS incidence and related outcomes.^24^ In supportive policy systems and enabling environments, these behaviors could be more readily adopted, contributing to improved functional ability.^20^ Thus, while macro-level factors like the Abuja Declaration are not shown to directly cause lower maternal mortality rates (MMRs), they would appear in the causal chain to set favorable conditions that ultimately reinforce individual actions and result in cumulative public health benefits.

**FIG. 1.**
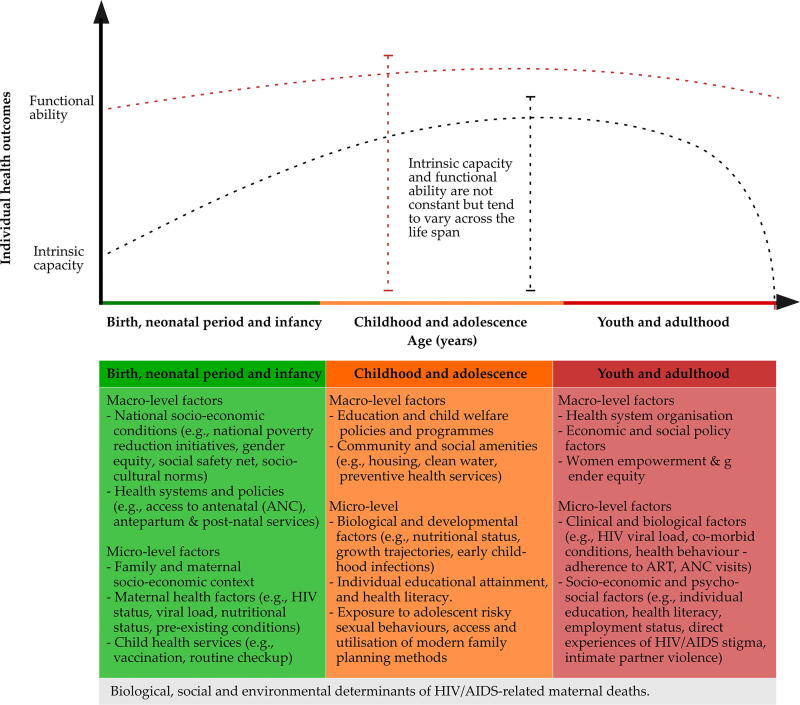
Conceptual framework showing biological, social, and environmental determinants of HIV/AIDS-related maternal deaths. This diagram depicts a life course model that illustrates how exposures and interventions during critical developmental periods (such as the neonatal period, early childhood, and adolescence) leave lasting imprints on women’s health. Essentially, these experiences shape both intrinsic capacity (the underlying biological reserves that determine a woman’s potential to maintain health) and functional ability, which is the capacity to utilize these reserves effectively in daily life through supportive environmental and social conditions. These biological, social, and environmental factors range from exposure to poor socioeconomic conditions (poverty) and HIV infections, to early access to preventive services and treatment, to quality maternal and child health services and subsequent social and environmental factors, which accumulate from birth through childhood, adolescence, and adulthood to shape maternal outcomes. Source: Adapted from Kuruvilla et al.^19^ HIV/AIDS, human immunodeficiency virus/acquired immunodeficiency syndrome.

In this study, we examined and compared current HIV/AIDS‐aggravated maternal deaths across 54 African countries, focusing on mortality trends over the past 39 years (with particular attention to shifts observed before the Abuja Declaration) and variations among different age groups and birth cohorts. In doing so, we aimed to derive evidence‐informed policy recommendations that can optimize interventions and contribute toward the Joint United Nations Programme on HIV and AIDS (UNAIDS) to eradicate the disease as a public health concern by 2030 (Fig. 1).^13^

### Study design

This serial ecological study was conducted as part of the Sub-Saharan Africa Research for Sustainable Development Project initiatives. The goals and objectives of this project are published elsewhere.^25^

### Study population and setting

The study population was women aged 15–49 years living in Africa. This age range was chosen to capture the standard reproductive years during which women are most likely to be pregnant and, consequently, at risk for maternal-related events, including death.^26^

Africa is one of the six global continents and the second most populous region after Asia. The continent comprises five subregions and 54 countries, each with unique socioeconomic characteristics.^27^ With approximately 1.4 billion people, Africa accounts for about 18% of the world’s population, which is predominantly youthful, with a median age of around 19 years.^28^ Females constitute nearly 50% of the total population, which is crucial in the context of HIV/AIDS-related maternal deaths in the region.^29^ The complete list of the countries included in this study is presented in Table 1.

**Table 1.** Number of HIV/AIDS-Aggravated Maternal Deaths, Age-Standardized Mortality Rate and Estimated Annual Percentage Change (Net Drift) Among the 54 Countries in Africa, 1992–2021

Subregion/	*N* mortalities (95% CI)	*N* mortalities (95% CI)	ASMR (95% CI)^a^	ASMR (95% CI)^a^	Net drift (95% CI)
Country	**1982**	**2021**	**1982**	**2021**	**1982–2021**
Central Africa					
Angola	0.03 (0, 0.11)	27.86 (12.4, 51.31)	∼0 (0, 0)	0.2 (0.09, 0.37)	10.01 (4.58, 15.71)
Cameroon	0.12 (0.03, 0.33)	50.33 (24.33, 79.6)	0 (0, 0.01)	0.35 (0.17, 0.54)	7.98 (5.35, 10.67)
CAR	∼0 (0, 0)	16.37 (6.89, 28.91)	∼0 (0, 0)	0.66 (0.28, 1.17)	8.3 (0.04, 17.23)
Chad	0.09 (0.02, 0.26)	22.73 (10.24, 39.6)	0 (0, 0.01)	0.34 (0.15, 0.59)	6.33 (3.38, 9.36)
Congo	0.01 (0, 0.03)	6.11 (2.63, 11.16)	∼0 (0, 0)	0.22 (0.1, 0.41)	2.04 (−0.95, 5.11)
DRC	7.08 (3.02, 15.4)	45.27 (21.89, 77.33)	0.05 (0.02, 0.11)	0.13 (0.06, 0.21)	−0.58 (−1.09, −0.08)
Equatorial Guinea	0 (0, 0.01)	3.43 (1.29, 7.12)	0 (0, 0.01)	0.5 (0.19, 1.04)	9.53 (−4.86, 26.1)
Gabon	0.01 (0, 0.02)	1.96 (0.83, 3.83)	0 (0, 0.01)	0.22 (0.09, 0.43)	5.71 (−1.54, 13.5)
São Tomé and Príncipe	∼0 (0, 0)	∼0 (0, 0)	∼0 (0, 0)	∼0 (0, 0)	−2.61 (−9.41, 4.71)
Eastern Africa					
Burundi	0.41 (0, 2.77)	7.63 (3.59, 13.74)	0.02 (0, 0.12)	0.15 (0.07, 0.27)	−2.52 (−3.82, −1.21)
Comoros	∼0 (0, 0)	∼0 (0, 0)	∼0 (0, 0)	∼0 (0, 0)	−2.3 (−9.13, 5.04)
Djibouti	∼0 (0, 0)	0.96 (0.36, 1.99)	∼0 (0, 0)	0.14 (0.05, 0.3)	2.02 (−4.03, 8.47)
Eritrea	0.13 (0.01, 0.71)	2.04 (0.79, 3.99)	0.01 (0, 0.06)	0.06 (0.02, 0.12)	−1.16 (−4.27, 2.06)
Ethiopia	0.76 (0.26, 1.67)	27.95 (13.67, 46.2)	0 (0, 0.01)	0.06 (0.03, 0.1)	0.01 (−0.91, 0.95)
Kenya	0.92 (0.46, 1.60)	62.06 (34.16, 100.49)	0.01 (0.01, 0.02)	0.28 (0.15, 0.45)	1.45 (0.69, 2.21)
Madagascar	∼0 (0, 0)	4.04 (1.19, 8.98)	∼0 (0, 0)	0.03 (0.01, 0.06)	11.68 (−0.5, 25.36)
Malawi	2.02 (0.28, 6.50)	48.81 (25.99, 79.13)	0.06 (0.01, 0.21)	0.59 (0.33, 0.94)	1.61 (0.88, 2.35)
Mauritius	∼0 (0, 0)	0.01 (0, 0.01)	∼0 (0, 0)	∼0 (0, 0)	11.59 (−86.5, 822.68)
Mozambique	0.19 (0.01, 0.71)	108.78 (59.8, 181.01)	0 (0, 0.01)	0.78 (0.42, 1.3)	10.62 (8.39, 12.89)
Rwanda	0.14 (0.01, 0.45)	12.36 (6.24, 19.94)	0.01 (0, 0.02)	0.2 (0.1, 0.32)	2.39 (0.36, 4.47)
Seychelles	∼0 (0, 0)	∼0 (0, 0)	∼0 (0, 0)	∼0 (0, 0)	−1.55 (−8.43, 5.85)
Somalia	∼0 (0, 0)	6.88 (2.08, 15.18)	∼0 (0, 0)	0.08 (0.02, 0.18)	13.25 (−6.42, 37.04)
South Sudan	0.05 (0, 0.22)	19.3 (5.1, 44.76)	0 (0, 0.01)	0.47 (0.12, 1.08)	8.35 (4.23, 12.63)
Tanzania	2.04 (0.59, 3.93)	136.22 (68.31, 222.81)	0.02 (0.01, 0.04)	0.52 (0.26, 0.84)	2.18 (1.62, 2.74)
Uganda	33.99 (5.39, 83.6)	70.14 (36.06, 112.27)	0.55 (0.08, 1.36)	0.39 (0.2, 0.63)	−2.53 (−2.89, −2.16)
Zambia	0.73 (0.09, 2.25)	40.52 (20.72, 66.11)	0.02 (0, 0.08)	0.46 (0.24, 0.73)	2.18 (1.06, 3.31)
Zimbabwe	0.8 (0.17, 1.9)	54.67 (26.78, 88.33)	0.02 (0, 0.05)	0.73 (0.36, 1.18)	4.64 (3.65, 5.65)
Northern Africa					
Algeria	0.01 (0.01, 0.02)	0.25 (0.12, 0.43)	∼0 (0, 0)	∼0 (0, 0)	6.6 (−20.9, 43.66)
Egypt	0 (0, 0.01)	0.05 (0.02, 0.12)	∼0 (0, 0)	∼0 (0, 0)	2.4 (−34.05, 59.01)
Libya	∼0 (0, 0)	0.01 (0, 0.04)	∼0 (0, 0)	∼0 (0, 0)	3.3 (−53.53, 129.62)
Morocco	0.03 (0.01, 0.08)	0.3 (0.13, 0.61)	∼0 (0, 0)	∼0 (0, 0)	1.81 (−7.98, 12.64)
Sudan	0.05 (0, 0.17)	4.05 (0.79, 12.04)	∼0 (0, 0)	0.02 (0, 0.05)	5.31 (−0.42, 11.37)
Tunisia	∼0 (0, 0)	0.01 (0, 0.01)	∼0 (0, 0)	∼0 (0, 0)	−3.01 (−11.57, 6.37)
Southern Africa					
Botswana	0.01 (0, 0.03)	1.74 (0.89, 2.77)	0 (0, 0.01)	0.12 (0.06, 0.2)	4.27 (−5.64, 15.21)
Eswatini	∼0 (0, 0)	2.63 (1.14, 4.83)	∼0 (0, 0)	0.45 (0.2, 0.82)	24.55 (−83.77, 856.02)
Lesotho	0.02 (0, 0.04)	8.9 (4.8, 14.73)	0 (0, 0.01)	0.95 (0.52, 1.53)	11.48 (4.87, 18.5)
Namibia	0.01 (0, 0.02)	2.8 (1.26, 5.26)	∼0 (0, 0)	0.23 (0.1, 0.43)	6.99 (−1.08, 15.71)
South Africa	0.07 (0.02, 0.14)	87.87 (61.9, 118.15)	∼0 (0, 0)	0.27 (0.19, 0.36)	12.84 (6.77, 19.26)
Western Africa					
Benin	∼0 (0, 0)	38.28 (16.92, 67)	∼0 (0, 0)	0.12 (0.05, 0.22)	15.58 (−26.87, 82.67)
Burkina Faso	1.51 (0.35, 3.43)	6.75 (2.88, 12.14)	0.04 (0.01, 0.1)	0.07 (0.03, 0.12)	−4.52 (−5.52, −3.51)
Cabo Verde	∼0 (0, 0)	6.32 (3.15, 11.4)	∼0 (0, 0)	0.01 (0, 0.02)	0.32 (−31.59, 47.12)
Cote d’Ivoire	0.91 (0.2, 2.05)	0.02 (0.01, 0.05)	0.02 (0.01, 0.05)	0.32 (0.14, 0.55)	0.5 (−0.31, 1.32)
Gambia	0 (0, 0.01)	3.57 (1.54, 6.89)	∼0 (0, 0)	0.33 (0.15, 0.64)	11.7 (−4.05, 30.04)
Ghana	0.34 (0.07, 0.91)	16.69 (7.8, 29.42)	0.01 (0, 0.02)	0.09 (0.04, 0.16)	3.17 (0.8, 5.6)
Guinea	0.06 (0.01, 0.15)	15.11 (6.73, 26.82)	0 (0, 0.01)	0.25 (0.11, 0.44)	7.77 (3.9, 11.78)
Guinea-Bissau	0.01 (0, 0.01)	2.59 (0.46, 6.8)	∼0 (0, 0)	0.25 (0.05, 0.66)	10.34 (−5.8, 29.25)
Liberia	0.02 (0, 0.07)	7.86 (3.56, 14.11)	0 (0, 0.01)	0.31 (0.14, 0.56)	7.3 (1.94, 12.94)
Mali	0.08 (0.01, 0.23)	15.93 (7.84, 28.82)	0 (0, 0.01)	0.17 (0.08, 0.3)	6.48 (2.96, 10.12)
Mauritania	∼0 (0, 0)	0.01 (0, 0.03)	∼0 (0, 0)	∼0 (0, 0)	4.12 (−46.81, 103.82)
Niger	0.04 (0.01, 0.12)	5.41 (2.18, 10.43)	∼0 (0, 0)	0.06 (0.03, 0.12)	4.61 (0.13, 9.28)
Nigeria	0.58 (0.25, 1.09)	151.28 (78.04, 267.08)	∼0 (0, 0)	0.15 (0.08, 0.27)	5.77 (4.71, 6.84)
Senegal	0.06 (0.02, 0.14)	5.31 (2.26, 10.28)	∼0 (0, 0)	0.08 (0.03, 0.15)	5.17 (0.97, 9.55)
Sierra Leone	0.02 (0, 0.07)	10.42 (4.7, 20.01)	∼0 (0, 0)	0.25 (0.12, 0.48)	10.04 (3.48, 17.01)
Togo	0.02 (0.01, 0.06)	5.58 (2.41, 9.93)	0 (0, 0.01)	0.14 (0.06, 0.24)	5.34 (0.09, 10.87)

^a^ Per 100,000 women.

ASIR, age-standardized incidence rate; CAR, Central African Republic; CI, confidence interval; DRC, Democratic Republic of Congo.

∼ means that the value is virtually zero when run to two decimal places.

Net drift represents the overall annual percentage change in the outcome measure (HIV/AIDS-aggravated maternal mortality), averaged over the entire study period, as estimated from the age-period-cohort (APC) model.

### Data source

This study utilized the Institute for Health Metrics and Evaluation (IHME) Global Burden of Disease Study 2021 (GBD 2021) data.^30^ For more than a decade, the IHME has consistently provided high-quality, regularly updated, and standardized datasets essential for global health monitoring, making the IHME the most comprehensive source of global health data. It obtains its data from a variety of sources, including vital registration systems (*e.g.,* WHO), health surveys, census data, hospital and clinical records, disease registries, scientific literature, and administrative data, making it the most comprehensive epidemiological study to date.^30^ While alternative data sources exist (*e.g.,* Global Health Observatory), many lack the same level of completeness, methodological transparency, and standardization required for our study; hence, we chose IHME.

### Variables

Annual age-specific, aggregate-level HIV/AIDS-aggravated maternal mortality and mid-year population data were extracted for each of the 54 African countries, spanning the period 1982–2021. Also, age-standardized mortality rate (ASMR) data were retrieved for the specified period. The period from 1982 to 2021 was selected for this study due to data availability. However, this timeframe also aligns with major developments in global and international health, including maternal and child health improvements and HIV/AIDS control in Africa.^31^

### Data analysis

We fitted Poisson regression-based APC models to the mortality (count) and population data to estimate HIV/AIDS-related maternal mortality trends and deviations for all 54 countries using the United States National Cancer Institute web tool for APC analysis.^32^ This statistical method is based on weighted least squares and the null assumption that the data followed a Poisson distribution. These functions are combinations of the age, period, and cohort parameters that the data can uniquely determine, irrespective of the nonidentifiability of their individual linear components.^32^

Since the mortality data extracted from the GBD 2021 database were available only in 5-year age groups, the same 5-year intervals were used for calendar periods and birth cohorts. We used seven 5-year age groups (15 through 19, 20 through 44, … 49 years) and eight 5-year time periods (1982 through 1986, 1987 through 1991, … 2017 through 2021), spanning 14 partially overlapping 5-year birth cohorts referred to by mid-year of birth (1937, 1942, … 2002). The central age group (30–34), calendar period (1997–2001), and birth cohort (1965–1969) were defined as the reference in all APC analyses to enhance interpretability and provide a stable benchmark against which all relative rate ratios (RR) are measured. We utilized the Tarone–Chu method to explore changes in APC period deviations circa 1999.5 (1997–2001), which marks the transition from pre- to post-Abuja summit eras. Specifically, we compared three 5-year periods before 1997–2001 (1982–1986, 1987–1991, and 1992–1996) with four 5-year periods after 1997–2001 (2002–2006, 2007–2011, 2012–2016, and 2017–2021).

Seven APC estimable and their corresponding 95% confidence intervals (CIs) were reported to capture the distinct facet of the APC structure underlying HIV/AIDS-aggravated maternal mortality.^32^ These included cross-sectional and longitudinal age curves (representing age effects), period RR, fitted temporal trends (representing period effects), cohort RR, and local drift (representing cohort effects), and net drift. In the context of this study:

- Net drift represents the overall annual percentage change in the outcome measure (HIV/AIDS-aggravated maternal mortality), averaged over the entire study period, as estimated from the APC model.
- Age effects represent the differences in maternal mortality risk associated with the biological and social processes of aging, independent of when a woman was born or the calendar period. For example, older maternal age is generally associated with higher obstetric risk, regardless of the year or cohort.
- Period effects are the changes that affect all age groups simultaneously due to events or interventions occurring at a specific time. In the context of this study, examples include the introduction of national HIV prevention programs, the rollout of ART, or continent-wide policy initiatives such as the Abuja Declaration in 2001.
- Cohort effects represent the differences in HIV/AIDS-aggravated maternal mortality attributable to the unique experiences of women born in the same time period (birth cohort), such as early-life exposure to HIV prevalence, nutrition, or health care access.

Given that when there is a progressive increase in rates from older to younger generations, the cross-sectional age curve tends to give the false impression of falling rates with advancing age at diagnosis (and vice versa), inferences about the age-related natural history of HIV/AIDS-related maternal mortality were made using the longitudinal age curve, which is not affected by this.^32^ In addition, we computed the test statistics for each parameter using the Wald test, which followed a chi-square distribution under the null hypothesis. To account for the risk of Type I errors arising from multiple comparisons in our APC analysis, Bonferroni correction was applied.^33^

We also performed a Joinpoint regression test to estimate the average annual percentage change (AAPC) for the period 1982–2019, thereby establishing a baseline trend excluding the coronavirus disease 2019 (COVID-19) pandemic years. Next, we extended the analysis to cover 1982–2021, which incorporates the COVID-19 years (2020–2021). This dual approach allowed us to compare the prepandemic trend with the extended trend that includes the pandemic years, providing insights into how COVID-19 may have influenced HIV/AIDS-related maternal mortality trends in Africa.

## Results

### Descriptive results

We estimated ASMR and the effects of age, calendar period, and birth cohorts on HIV/AIDS-aggravated maternal mortality, separately for each of the 54 African countries. There were 47,750 maternal deaths with 8.52 × 10^9^ woman years of follow-up among women aged 15–49 years, from 1982 through 2021. In line with the objectives of this study, we compared the most recent (2021) HIV/AIDS-aggravated MMR, as measured by ASMR per 100,000 women, among the 54 countries. We found that in 2021, 16 of the 54 countries had ASMRs near 0, while 38 countries reported rates ranging from 0.07 to 0.95 per 100,000 women (Table 1).

Notably, five out of the six Northern African countries recorded ASMRs of 0, implying that no HIV/AIDS-aggravated maternal deaths were detected in these countries. However, in Eastern Africa, Kenya and Uganda recorded moderate rates of approximately 0.28 and 0.39, whereas Mozambique, Malawi, and Zimbabwe reached higher figures of around 0.78, 0.59, and 0.73 per 100,000 women, respectively. Similarly, in Central Africa, the Central African Republic reported an ASMR of about 0.66, with Cameroon and Chad recording approximately 0.35 and 0.34, respectively. Southern African countries showed mixed results; South Africa reported a moderate rate of about 0.27, while Lesotho recorded the highest in the region, at nearly 0.95, even though Botswana, Eswatini, and Namibia maintained near-zero rates. In Western Africa, Mauritania reported an ASMR of exactly 0, but the other 15 countries had values between 0.01 and 0.33 (Table 1).

### APC modeling results

#### Period effects

**FIG. 2.**
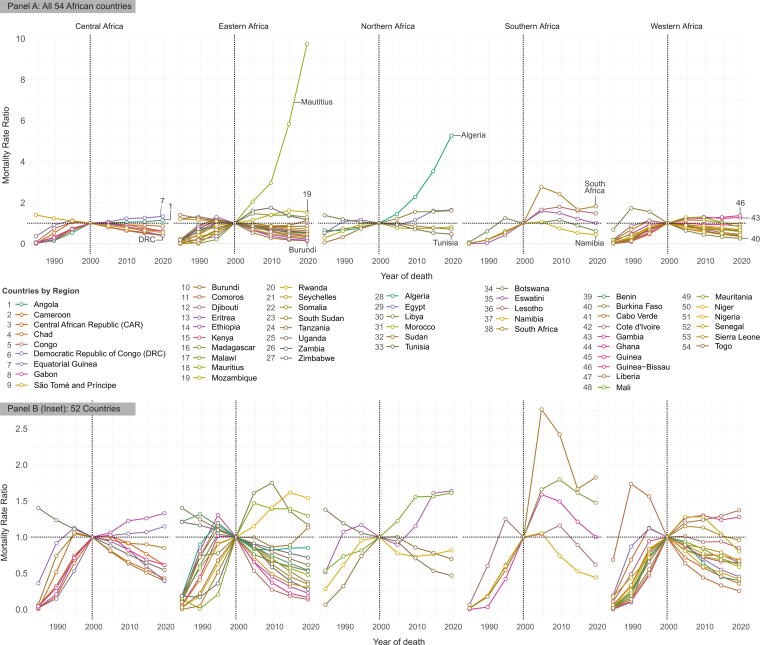
Period rate ratios (RRs) of age‐specific HIV/AIDS-aggravated maternal mortality relative to 1999.5 (year before the Abuja Summit). The horizontal axis (X-axis) represents the calendar period, while the vertical axis (Y-axis) displays the RRs for a specific calendar period within an individual country, and the connecting lines show the overall period-related trend. Different colors distinguish the trends for the 54 African countries, highlighting variations in the trajectory of MMR. A *horizontal dotted line* at 1.0 marks the reference level; values above 1.0 indicate that, in that particular period, maternal mortality rate (MMR) was higher relative to the reference period (1999.5). On the contrary, values below 1.0 denote that the MMR was lower compared with that of 1999.5. This figure, representing period effects, illustrates changes that affect all age groups simultaneously due to events or interventions occurring at a specific time. In the context of this study, examples include the introduction of national HIV prevention programs, the rollout of ART, or continent-wide policy initiatives such as the Abuja Declaration in 2001.

We examined the evolution of HIV/AIDS-aggravated maternal mortality in Africa from 1982 to 2021, using the Abuja Summit reference period (1999.5, RR = 1) to compare pre- and postsummit trends. Our analysis focused on whether country-specific mortalities increased or decreased relative to that benchmark (Fig. 2).

In Central Africa, Angola’s RR rose from just 0.02 in 1984.5 (98% below the benchmark) to 1.15 by 2019.5, indicating a transition from exceptionally low to modestly elevated mortality. The Democratic Republic of Congo (DRC) followed a different path: it reached the benchmark in 1999.5 then fell to 0.40 by 2019.5, 60% below the reference (Fig. 2).

Eastern Africa saw many countries rising toward RR = 1 before declining postsummit. Tanzania’s ratio increased from 0.19 in 1984.5 to 1.20 by the mid-1990s, then fell to 0.85 in 2019.5. Uganda matched the benchmark in 1999.5 but dropped to 0.54 by 2019.5, reflecting a nearly 46% reduction relative to the summit period. In what appeared to be a direct contrast, Mauritius showed a sustained and steep increase, rising from 0.24 in 1984.5 to 9.75 in 2019.5, with no postsummit decline.

Northern Africa displayed heterogeneous shifts. Algeria’s RR grew substantially, from 0.66 in 1984.5 to 5.27 in 2019.5, over 400% above the summit level, while Morocco increased from 0.28 to the benchmark by 1999.5 and then stabilized around 0.82 in 2019.5, about 18% below RR = 1.

In Southern Africa, Botswana rose from 0.07 in 1984.5 to the benchmark in 1999.5 before declining to 0.62 by 2019.5. South Africa’s mortality surged from 0.01 in 1984.5 to RR = 1 in 1999.5, peaked at 2.77 in 2004.5, and then receded to 1.82 in 2019.5, remaining well above the reference.

Western Africa’s postsummit paths were equally varied. Burkina Faso reached RR = 1 in 1999.5, then plunged to 0.26 by 2019.5 (74% below the benchmark), whereas Guinea-Bissau rose from 0.02 in 1984.5 to the summit level and further to 1.37 in 2019.5, 37% above the reference (Fig. 2).

Our model-derived fitted temporal trends (Supplementary Fig. S1) further indicate that MMRs were very low in the early 1980s, but increased markedly, reaching high levels during the 1990s and early 2000s in several regions. The Wald test results for period deviations suggest that while some countries (25/54) experienced a steady exponential increase in maternal mortality over time, the majority showed more complex, nonlinear trajectories, even after considering age and cohort effects (Table 2).

**Table 2.** Summary of the Wald Test Results Illustrating Whether the Estimated Age, Period, and Cohort Deviation Parameters Differ Significantly from Zero, Suggesting Nonlinear Variation Beyond the Overall Linear Trend (Net Drift)

Country by region	Age deviations			Period deviations			Cohort deviations		
	***χ*** ^2^	df	*P*	**χ** ^2^	df	*p*	**χ** ^2^	df	*p*
Central Africa									
Angola	64.9	5	<0.0001	16.08	12	0.1874	26.84	6	0.0002
Cameroon	153.04	5	<0.0001	38.06	12	0.0002	145.37	6	<0.0001
CAR	127.08	5	<0.0001	15.54	12	0.2133	49.49	6	<0.0001
Chad	60.39	5	<0.0001	20.26	12	0.0624	33.35	6	<0.0001
Congo	34.56	5	<0.0001	5.53	12	0.938	22.68	6	0.0009
DRC	312.86	5	<0.0001	91.51	12	<0.0001	189.9	6	<0.0001
Equatorial Guinea	6.37	5	0.272	0.82	12	0.9999	1.99	6	0.9203
Gabon	6.95	5	0.2242	1.64	12	0.9998	6.89	6	0.3309
São Tomé and Príncipe	0	5	0.9999	0.03	12	0.9999	0	6	0.9999
Eastern Africa									
Burundi	118.84	5	<0.0001	36.77	12	0.0002	181.63	6	<0.0001
Comoros	0	5	0.9999	0.02	12	0.9999	0	6	0.9999
Djibouti	7.47	5	0.1878	1.89	12	0.9996	5.02	6	0.5416
Eritrea	39.76	5	<0.0001	7.13	12	0.849	29.57	6	<0.0001
Ethiopia	320.66	5	<0.0001	99.82	12	<0.0001	503.6	6	<0.0001
Kenya	263.57	5	<0.0001	153.5	12	<0.0001	318.6	6	<0.0001
Madagascar	11.64	5	0.0401	1.15	12	0.9999	6.21	6	0.4
Malawi	150.43	5	<0.0001	49.55	12	<0.0001	169.47	6	<0.0001
Mauritius	0.02	5	0.9999	0	12	0.9999	0	6	0.9999
Mozambique	182.43	5	<0.0001	35.47	12	0.0004	109.02	6	<0.0001
Rwanda	65.76	5	<0.0001	25.98	12	0.0108	98.6	6	<0.0001
Seychelles	0	5	0.9999	0.2	12	0.9999	0	6	0.9999
Somalia	44.26	5	<0.0001	7.13	12	0.8488	37.27	6	<0.0001
South Sudan	67.2	5	<0.0001	8.01	12	0.7842	30.42	6	<0.0001
Tanzania	368.1	5	<0.0001	170.6	12	<0.0001	194.63	6	<0.0001
Uganda	414.95	5	<0.0001	64.12	12	<0.0001	10.8	6	0.0946
Zambia	144.91	5	<0.0001	38.77	12	0.0001	124.66	6	<0.0001
Zimbabwe	115.94	5	<0.0001	31.74	12	0.0015	150.52	6	<0.0001
Northern Africa									
Algeria	0.5	5	0.992	0.05	12	0.9999	0.02	6	0.9999
Egypt	0.06	5	0.9999	0.02	12	0.9999	0.02	6	0.9999
Libya	0.02	5	0.9999	0	12	0.9999	0	6	0.9999
Morocco	1.47	5	0.9168	0.37	12	0.9999	0.41	6	0.9988
Sudan	8.52	5	0.1299	1.38	12	0.9999	10.79	6	0.095
Tunisia	0.04	5	0.9999	0.18	12	0.9999	0.03	6	0.9999
Southern Africa									
Botswana	9.56	5	0.0886	1.51	12	0.9999	7.72	6	0.2593
Eswatini	2.95	5	0.7081	1.09	12	0.9999	11.84	6	0.0657
Lesotho	16.37	5	0.0059	3.54	12	0.9904	27.08	6	0.0001
Namibia	5.08	5	0.4056	2.99	12	0.9956	19.5	6	0.0034
South Africa	174.9	5	<0.0001	69.05	12	<0.0001	229.19	6	<0.0001
Western Africa									
Benin	27.33	5	<0.0001	9.14	12	0.6908	11.75	6	0.0678
Burkina Faso	59.75	5	<0.0001	19.41	12	0.0791	51.81	6	<0.0001
Cabo Verde	0.4	5	0.9952	0.07	12	0.9999	0.16	6	0.9999
Cote d’Ivoire	193.38	5	<0.0001	50.66	12	<0.0001	119.64	6	<0.0001
Gambia	5.7	5	0.3368	2.09	12	0.9993	5.22	6	0.5162
Ghana	100.46	5	<0.0001	12.21	12	0.4288	35.25	6	<0.0001
Guinea	32.01	5	<0.0001	13.77	12	0.3154	29.58	6	<0.0001
Guinea-Bissau	10.53	5	0.0615	1.06	12	0.9999	3.1	6	0.7967
Liberia	24.52	5	0.0002	5.41	12	0.9427	20.01	6	0.0028
Mali	67.44	5	<0.0001	15.94	12	0.194	43.74	6	<0.0001
Mauritania	0.01	5	0.9999	0.03	12	0.9999	0.02	6	0.9999
Niger	27.45	5	<0.0001	9.27	12	0.6796	23.98	6	0.0005
Nigeria	454.79	5	<0.0001	172.59	12	<0.0001	308.24	6	<0.0001
Senegal	20.29	5	0.0011	8.68	12	0.7303	14.04	6	0.0292
Sierra Leone	29.13	5	<0.0001	4.52	12	0.9721	22	6	0.0012
Togo	34.03	5	<0.0001	8.47	12	0.7476	32.22	6	<0.0001

*p* < 0.00093 is considered statistically significant, meaning that the Null Hypothesis (below) is unlikely to be true. Original α = 0.05; Bonferroni-adjusted α’ was calculated as 0.05/54 = 0.00093.

Interpretation: A significant result (*p* < 0.00093) suggests that the corresponding effect exhibits curvature that cannot be explained by a simple linear change over time.

CAR, Central African Republic; DRC, Democratic Republic of Congo; ST, São Tomé and Príncipe; df, degree of freedom.

#### Age effects

**FIG. 3.**
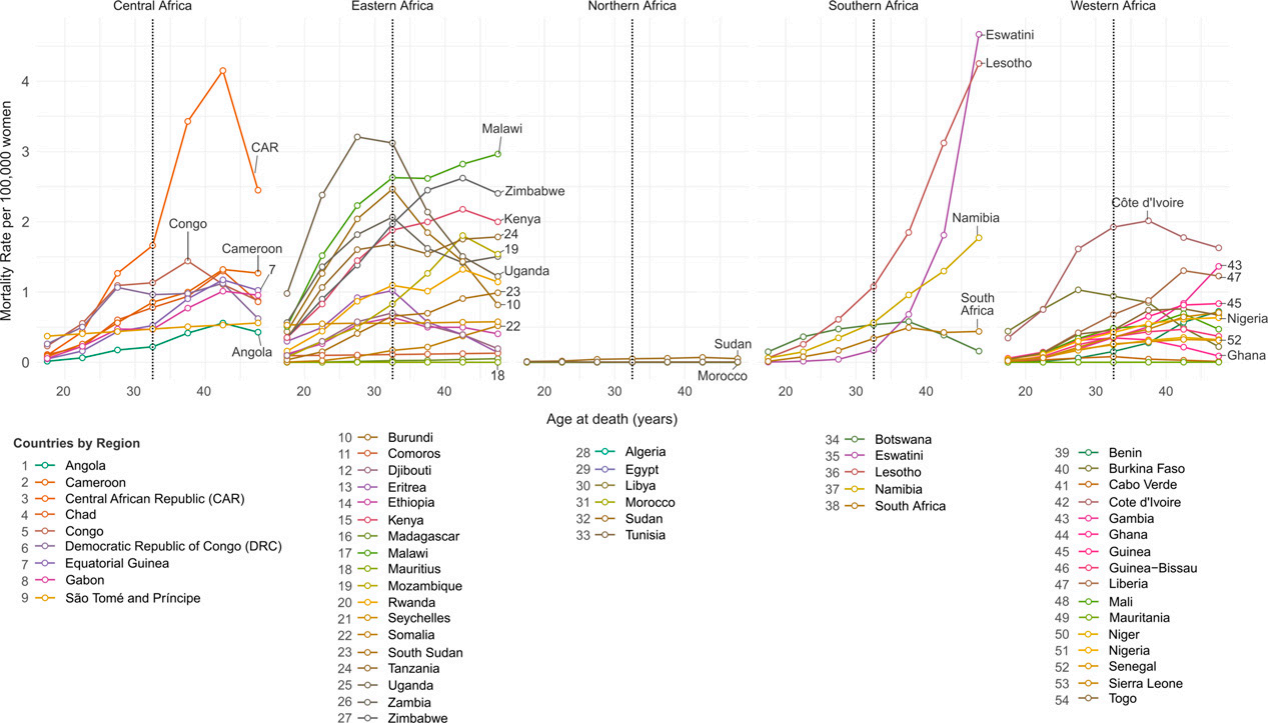
Longitudinal age curve showing expected age‐specific HIV/AIDS-aggravated maternal mortality rate (MMR) across a defined age range, adjusted for period effects. The horizontal axis (X-axis) represents the age groups, while the vertical axis (Y-axis) displays the expected MMR values. Each point on the curve corresponds to the estimated MMR for women at that specific age, and the connecting line highlights the overall age‐related trend. Different colors distinguish curves for various countries, highlighting variations in the trajectory and magnitude of MMR across diverse settings in Africa. This figure, representing age effects, highlights the differences in maternal mortality risk associated with the biological and social processes of ageing, independent of when a woman was born or the calendar period. For example, older maternal age is generally associated with higher obstetric risk, regardless of the year or cohort.

Our age-specific analyses reveal distinct age patterns in MMR trajectories across Africa, with the shape and steepness of the rate curves varying markedly between and within regions (Fig. 3).

In Central Africa, Angola’s maternal mortality builds gradually, from 0.02 per 100,000 at age 17.5 to 0.56 by age 42.5, then dips slightly, whereas Cameroon’s curve rises sharply from 0.08 to 1.32 before a modest decline, illustrating divergent age-related risk accumulation even within the same region.

Eastern Africa shows contrasting patterns. For instance, Kenya’s MMR increases nearly linearly from 0.35 at 17.5 to 2.18 at 42.5 before tapering, while Uganda peaks early, jumping from 0.98 at 17.5 to 3.21 at 27.5 and then falling to 1.22 by 47.5, suggesting a younger age burden in Uganda versus a steady risk rise in Kenya.

In Northern Africa, Sudan’s rates rose slowly from 0.01 at age 17.5 to 0.07 at 42.5 before leveling, but Morocco maintains extremely low MMRs throughout (from 0.001 to 0.01, then back toward 0), indicating minimal cumulative mortality with age.

Southern Africa’s curves diverge widely, with Lesotho showing a dramatic surge from 0.06 at 17.5 to over 4.25 at 47.5, whereas South Africa’s curve is much flatter, rising from 0.01 to around 0.49 and settling near 0.44, reflecting a less pronounced age effect.

Western Africa also varies: Nigeria’s risk increased moderately from 0.05 at 17.5 to 0.64 by 47.5, while Côte d’Ivoire starts at 0.35, peaks at 2.01 by 37.5, then declines slightly, indicating a steeper age-related increase in HIV/AIDS-aggravated maternal mortality (Fig. 3).

These results are consistent with the cross-sectional age curve, which shows similar increasing trends with age (Supplementary Fig. S2). Like the longitudinal age curve, the 95% CIs widen with age, indicating greater uncertainty in estimates for older age groups. However, none of the CIs include zero, suggesting that the true age effect is statistically significant. The Wald test results for age deviations further suggest that while some countries experience a steady exponential increase in age-specific maternal mortality, the majority (31/54) exhibit more complex, nonlinear age patterns, even after adjusting for period and cohort effects (Table 2).

#### Cohort effects

**FIG. 4.**
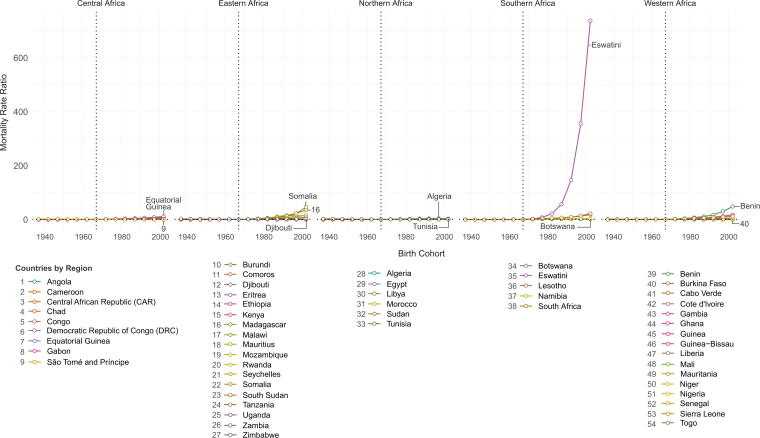
Cohort rate ratios (RRs) of age‐specific HIV/AIDS-aggravated maternal mortality relative to the 1967 birth cohort (the central cohort defined as the reference in all our age-period-cohort modeling). The horizontal axis (X-axis) represents the birth cohort, while the vertical axis (y-axis) displays the RRs for a specific birth cohort within an individual country, and the connecting lines show the overall age-related trend. Different colors distinguish the trends for the 54 African countries, highlighting variations in the trajectory of MMR. A horizontal dotted line at 1.0 marks the reference level; values above 1.0 indicate that, in that particular cohort, maternal mortality rate (MMR) is higher relative to the reference cohort (1967). Conversely, values below 1.0 denote that the MMR was lower compared with that of 1967. This figure, representing cohort effects, shows the differences in HIV/AIDS-aggravated maternal mortalities attributable to the unique experiences of women born in the same time period (birth cohort), such as early-life exposure to HIV prevalence, nutrition, or health care access.

Our cohort analyses compare age-specific HIV/AIDS-related maternal mortality ratios for each birth cohort against the 1967 reference (RR = 1). Although the earliest and some later cohorts have wide confidence intervals, most likely due to sparse data, distinct generational trends emerge, highlighting substantial regional diversity in maternal mortality trajectories across Africa (Fig. 4).

In Central Africa, Angola and Cameroon show sharply rising cohort RR. Women born in 1937 in Angola had an RR of about 0.03, which increased nearly 10-fold by the 2002 cohort. Cameroon shows a similar but somewhat less steep increase, from roughly 0.05 in 1937 to about 5.6 by 2002.

Eastern Africa presents two contrasting patterns. In South Sudan, extremely low early-cohort RRs (with high uncertainty) give way to values around 7.6 for the 2002 cohort. By contrast, Uganda’s RRs decline steadily from near 1 in early cohorts to as low as 0.15 in the 2002 cohort.

Northern Africa shows mixed generational effects compounded by imprecision. Sudan’s cohort RRs gradually rise from very low early values to several times the 1967 benchmark by 2002, whereas Tunisia’s later cohorts consistently exhibit lower RRs despite wide early confidence intervals.

Southern Africa also demonstrates heterogeneity in cohort trends. South Africa and Botswana display gradual increases from low early-cohort RRs to markedly higher values in recent generations, while Lesotho and Eswatini experience even steeper rises.

Western Africa arguably shows the greatest diversity. Whereas Guinea-Bissau’s generational effect is explosive, reaching an estimated 12.7 in the 2002 cohort, Ghana’s cohort RRs increased moderately from about 0.31 for women born in the 1930s to 1.36 by 2002. Other countries such as Burkina Faso and Mali show either modest increases or slight declines (Fig. 4).

These results are consistent with the local drift, which shows similar divergent patterns of HIV/AIDS-related maternal mortality among the different birth cohorts in Africa (Supplementary Fig. S3). The Wald test results for cohort deviations suggest that while many countries (39/54) exhibit a steady, log-linear increase, 15 out of 54 show significant nonlinearity in maternal mortality across successive birth cohorts (Table 1).

##### Sensitivity analysis

Our Joinpoint regression analysis indicates that the inclusion of the COVID-19 years results in only modest differences in the AAPC estimates across the 54 African countries studied. For example, in Angola, we observed an AAPC of 15.22% for 1982–2019, which slightly decreased to 14.28% for 1982–2021. Similarly, for Uganda, the AAPC shifted from –1.14% during 1982–2019 to –1.39% when the COVID-19 period was included (Supplementary Fig. S4). These minor variations, which were consistent across other countries, suggest that the overall direction and significance of the trends remain robust regardless of whether the COVID-19 pandemic years are part of the analysis.

## Discussion

### Trends and disparities in HIV/AIDS-related maternal deaths

This study examined and compared current HIV/AIDS‐aggravated maternal deaths across 54 African countries, assessed mortality trends over 39 years with a focus on shifts observed before and after the Abuja Declaration, and explored variations among different age groups and birth cohorts. Our analysis revealed significant regional- and country-level disparities: many Northern and Western African countries recorded near‐zero rates, while several Eastern, Central, and Southern nations reported relatively higher rates. Period analyses showed that although many countries had converged to the Abuja benchmark by the late 1990s, their trajectories diverged thereafter, with some, such as Angola, experiencing exponential increases and others, such as the DRC and Uganda, showing notable declines. Notably, Mauritius and Algeria exhibited trajectories that diverged substantially from the general continental pattern, with sustained increases rather than the postsummit declines seen in many countries. Age‐specific findings demonstrated that mortality generally increased with age, but the patterns differed markedly, as seen in Kenya’s near‐linear rise versus Uganda’s pronounced early peak. Cohort analyses further revealed that, in many regions, recent birth cohorts exhibited considerably higher mortality relative to the 1967 reference.

These findings indicate that the burden of HIV/AIDS‐aggravated maternal mortality varies considerably among African countries, age groups, and birth cohorts. For example, while Algeria, Mauritius, Angola, and Lesotho experienced notable increases over time, Uganda and DRC exhibited declines post Abuja Summit. Although it is not clear what might have caused this, the persistent upward trajectories for Algeria and Mauritius suggest that country-specific factors, such as the differences in health care access, the scale and timeliness of ART roll-out, and the implementation of maternal health interventions, could play a role. Learning from other African countries, early and expanded ART coverage in Uganda has been linked to improved maternal outcomes, as reported by UNAIDS,^34^ whereas slower intervention scale-up in Angola and Lesotho might have contributed to their rising trends.^35^ Similar comparative insights could help interpret divergent patterns in Algeria and Mauritius, underscoring the importance of timely ART initiation and sustained program coverage for preventing HIV/AIDS-aggravated maternal mortality.

Similarly, the age‐specific patterns, such as the steep increase in mortality observed in Cameroon compared with the gradual rise in Angola, could reflect variations in cumulative risk factors, possibly due to differences in the management of comorbidities or variations in long-term exposure to HIV-related complications. Deaney and colleagues argue that disparities in routine care and sustained healthcare engagement can influence how risks accumulate among marginalized populations over time, providing support for our observations.^36^ Their findings suggest that staggered implementation of health policies can exacerbate inequalities in rural and marginalized communities. While our interpretations are consistent with this evidence, it is also important to acknowledge alternative explanations, such as data quality issues or unmeasured social determinants, which may have contributed to the observed differences.^36^ For example, variations in data quality and collection methods across different countries have been documented and could have contributed to the wide confidence intervals and divergent trends seen in settings such as the pronounced cohort effect in Guinea‐Bissau.^37,38^

Furthermore, our cohort analyses revealed that later-born cohorts faced significantly higher mortality ratios relative to the 1967 reference. A possible explanation for this trend is the epidemiological transition going on in Africa, with changes in early-life exposures and even the potential emergence of drug-resistant HIV strains,^39–41^ a phenomenon also noted by the WHO.^42^ Moreover, differences in the timing and scale of health policy implementation, including the roll-out of ARTs and maternal health services, might partially explain the contrasting period and age effects observed between countries such as South Africa and Tunisia.^43^

### Policy implications and recommendations

**FIG. 5.**
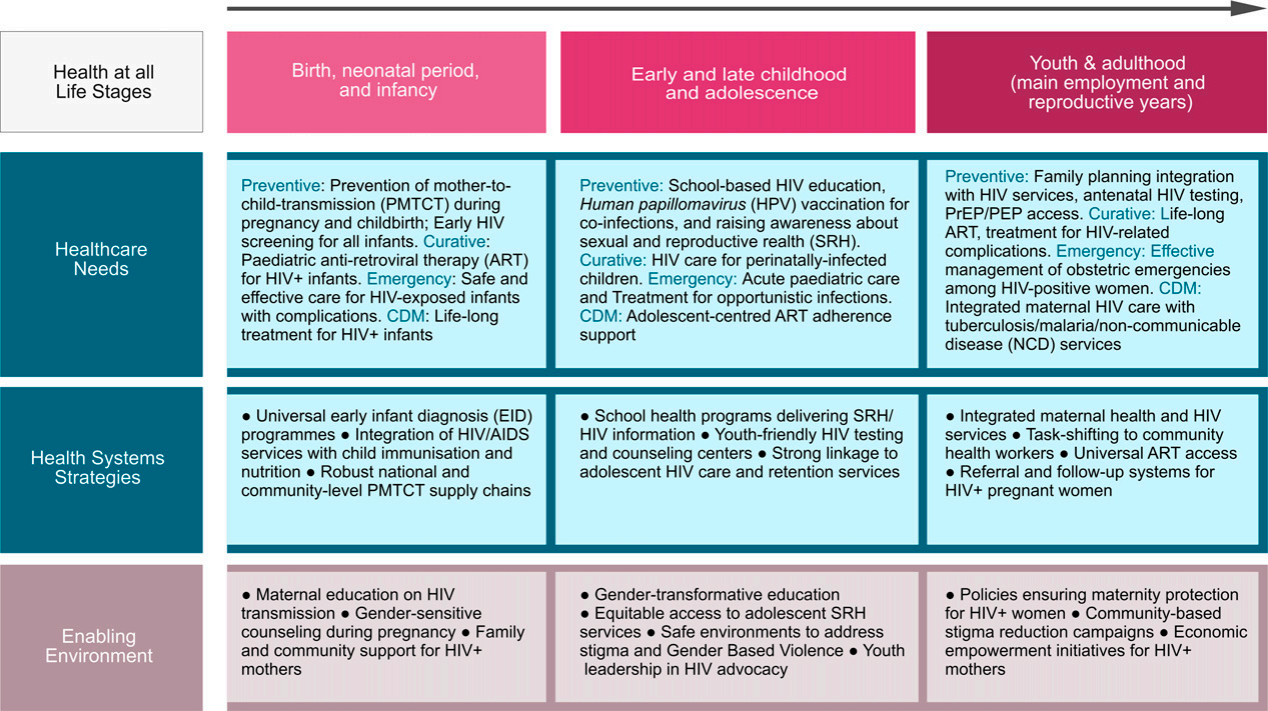
Framework for planning universal health coverage for HIV/AIDS control using a life-course approach. This diagram outlines HIV interventions across three life stages, birth/neonatal/infancy, childhood/adolescence, and youth/adulthood by organizing health care needs into four domains: preventive (*e.g.,* PMTCT, early screening, educational initiatives, and vaccination), curative (*e.g.,* pediatric and lifelong ART, targeted HIV care, and treatment for complications), emergency (*e.g.,* neonatal and obstetric care), and chronic disease management (*e.g.,* early diagnosis and integrated lifelong treatment). It also highlights key health system strategies (such as universal early infant diagnosis, service integration, youth-friendly centers, and robust referral systems) alongside an enabling environment characterized by maternal education, gender-sensitive counseling, community support, and stigma reduction, with reference numbers indicating supporting sources. PrEP/PEP, pre-exposure prophylaxis and postexposure prophylaxis. While this figure was not derived directly from the present analysis, it aligns well with the trends observed, and is based on the synthesis of key policy recommendations from the literature and established international policy guidelines, presented here to contextualize the study’s findings and illustrate potential strategies for reducing HIV/AIDS-related maternal mortality.^5,13,34,45–57^

Our study findings have important implications for designing universal health coverage (UHC) strategies for HIV/AIDS control, using a life‐course approach that addresses the unique health challenges of different demographic groups and geographical contexts (Fig. 5). Our analysis has shown that HIV/AIDS‐aggravated maternal mortality varies substantially across countries, with Angola and Lesotho experiencing increasing mortality trends, whereas Uganda and DRC reported declines following the Abuja Declaration. This variation suggests that a one‐size‐fits‐all strategy is unlikely to be effective. Instead, targeted, context-specific policies should be implemented.

In settings such as Angola and Lesotho, where rising MMRs are particularly pronounced, poor early-life interventions may be a contributing factor, especially among newborns and infants. Here, strengthening and scaling up universal PMTCT programs and early infant diagnosis (EID) is critical.^35^ Existing evidence shows that countries such as Uganda, which benefited from early and wide rollout of ART and robust PMTCT services, have subsequently experienced declines in maternal mortality.^34,44^ Similarly, Angola and Lesotho could benefit from intensified efforts to improve pediatric ART services, integrated neonatal care, and the establishment of reliable supply chains that link infant immunization and nutrition with routine HIV screening.^34^

Our study also revealed distinct age‐related mortality patterns. For example, in Cameroon, a steep rise in mortality with age suggests that cumulative risk factors are not being adequately addressed in older women. To address this, policymakers should consider implementing integrated postpartum care models that combine maternal HIV care with routine screening and treatment for comorbidities, such as TB, malaria, and noncommunicable diseases, as well as expand chronic disease management services.^45,46^ In contrast, settings that show higher mortality among younger women, as observed in Uganda, may require policies that expand school‐ and community‐based HIV education, create youth-friendly testing and counseling centers, and ensure that interventions such as pre-exposure prophylaxis/postexposure prophylaxis are widely available.^47,48^

Furthermore, our cohort analysis showed that in countries such as Angola and Cameroon, recent birth cohorts experienced a higher mortality rate relative to the 1967 reference group, potentially reflecting emerging risks/exposures and systemic shifts in health care delivery, access, and quality. For countries facing rising mortality among recent cohorts, scaling up early interventions, including universal PMTCT, prompt pediatric ART initiation, and effective linkage to community-based HIV services, is paramount.^49,50^ Conversely, where cohorts are showing a declining trend, as in Uganda, ongoing monitoring and continued investment in current programs are essential to sustain and further improve these positive outcomes.^6,24^

Finally, in settings such as Guinea-Bissau, where our analysis revealed an explosive cohort effect, the need to improve data quality and enhance national surveillance systems is urgent.^20^ Strengthening these areas can enable more accurate targeting of resources and interventions. In addition, adopting community-level strategies such as task shifting to community health workers,^51^ and implementing supportive policies such as maternity protection for HIV-positive women are crucial for addressing both clinical and social determinants of maternal mortality.^52^

Figure 5 provides a synthesis of key policy recommendations from the literature and established international policy guidelines, presented here to contextualize the study’s findings and illustrate potential strategies for reducing HIV/AIDS-related maternal mortality.^5,13,34,45–57^ These recommendations are not derived directly from the present analysis but are aligned with the trends observed.

### Strengths and limitations

This study’s main strengths lie in its comprehensive scope and multidimensional analytical framework. By covering 54 countries over 39 years, the study offers unparalleled insight into age, period, and cohort trends in HIV/AIDS-related maternal deaths across diverse settings. We utilized the GBD 2021 dataset, which provides a comprehensive and harmonized source of data on global health metrics. Employing established benchmarks such as the Abuja Declaration further added a strong policy-relevance dimension to the study.

Despite the insightful findings, some limitations persist. The observational and cross-sectional nature of the study precludes definitive causal inferences. Additionally, it could be useful to provide country-specific contextual data (*e.g.,* health care access, ART rollout timelines, and other interventions), which would enhance interpretation. However, given the breadth of the dataset (covering 39 years and 54 African countries), it is not feasible to provide detailed contextualization for each country within the scope of this article. That said, we believe that the study’s strength lies in its comprehensive, continent-wide scope and long-term temporal coverage, which allow for the identification of broad patterns and evolutions in HIV/AIDS-related maternal mortality over time. This provides a valuable benchmark for future, more context-specific investigations.

Future longitudinal studies using individual-level data could provide deeper insights into causal pathways and better capture the impact of specific health interventions over time. Additionally, future analyses should incorporate comprehensive socio-economic and local policy indicators to elucidate the underlying factors driving changes in HIV/AIDS‐related maternal mortality. Such efforts will be essential for designing context-specific, life-course strategies that more effectively inform UHC and targeted public health interventions in Africa.

## Conclusions

Two decades and counting since Africa’s historic Abuja Summit, the fight against HIV/AIDS and maternal mortality remains as critical as ever. In this study, we analyzed HIV/AIDS‐related maternal deaths across 54 African countries over 39 years using the GBD 2021 dataset, with a particular interest in understanding cross-national comparisons, temporal trends (highlighting important changes before and after the Abuja period), and variations across age groups and birth cohorts. Our analysis revealed notable variation in HIV/AIDS‐aggravated maternal mortality across Africa. In 2021, 16 of the 54 countries had ASMRs near zero, while 38 countries reported rates ranging from 0.07 to 0.95 per 100,000 women. Mortality rates rose sharply from the early 1980s, peaked during the 1990s and early 2000s, and generally declined after the Abuja Summit, although trajectories varied considerably across the continent. Mortality also increased with age, with substantial heterogeneity in country-level patterns. In more than two-thirds of countries, cohort comparisons relative to a 1967 baseline showed marked increases in mortalities in recent birth cohorts. These findings indicate that the burden of HIV/AIDS‐aggravated maternal mortality varies considerably among African countries, age groups, and birth cohorts. In line with these findings, our study supports the need for context-specific, life-course strategies, ranging from strengthened PMTCT and EID services to integrated maternal care and youth-targeted HIV education, to drive progress toward UHC and reduce maternal mortality across Africa.

## Supplementary Material

Supplementary Figure S1

Supplementary Figure S2

Supplementary Figure S3

Supplementary Figure S4

## Data Availability

The original data presented in the study are openly available in publicly accessible repositories (https://vizhub.healthdata.org/gbd-results/).

## Abbreviations Used

AAPC: average annual percentage change
APC: age-period-cohort
ART: antiretroviral treatment
ASMR: age-standardized mortality rate
CI: confidence interval
COVID-19: coronavirus disease 2019
DRC: Democratic Republic of Congo
EID: early infant diagnosis
GBD: Global Burden of Disease
HIV/AIDS: human immunodeficiency virus/acquired immunodeficiency syndrome
IHME: Institute for Health Metrics and Evaluation
MMR: maternal mortality rate
PMTCT: prevention of mother-to-child transmission
OAU: Organisation of African Unity
ODA: official development assistance
RR: rate ratio
TB: tuberculosis
UHC: Universal Health Coverage
UNAIDS: United Nations Programme on HIV and AIDS
WHO: World Health Organization
