# Supplementary material for: Two Decades and Counting Since the Abuja Summit: Where Do We Stand in the Fight Against HIV/AIDS-Related Maternal Mortality?
Source: Womens Health Rep (New Rochelle). 2025 Oct 8;6(1):1092–108. doi: 10.1177/26884844251386289 (PMC12549178; doi:10.1177/26884844251386289)
Supplement: Supplementary Figure S1 [file 26884844251386289_supplementary_figure_s1.docx]

**
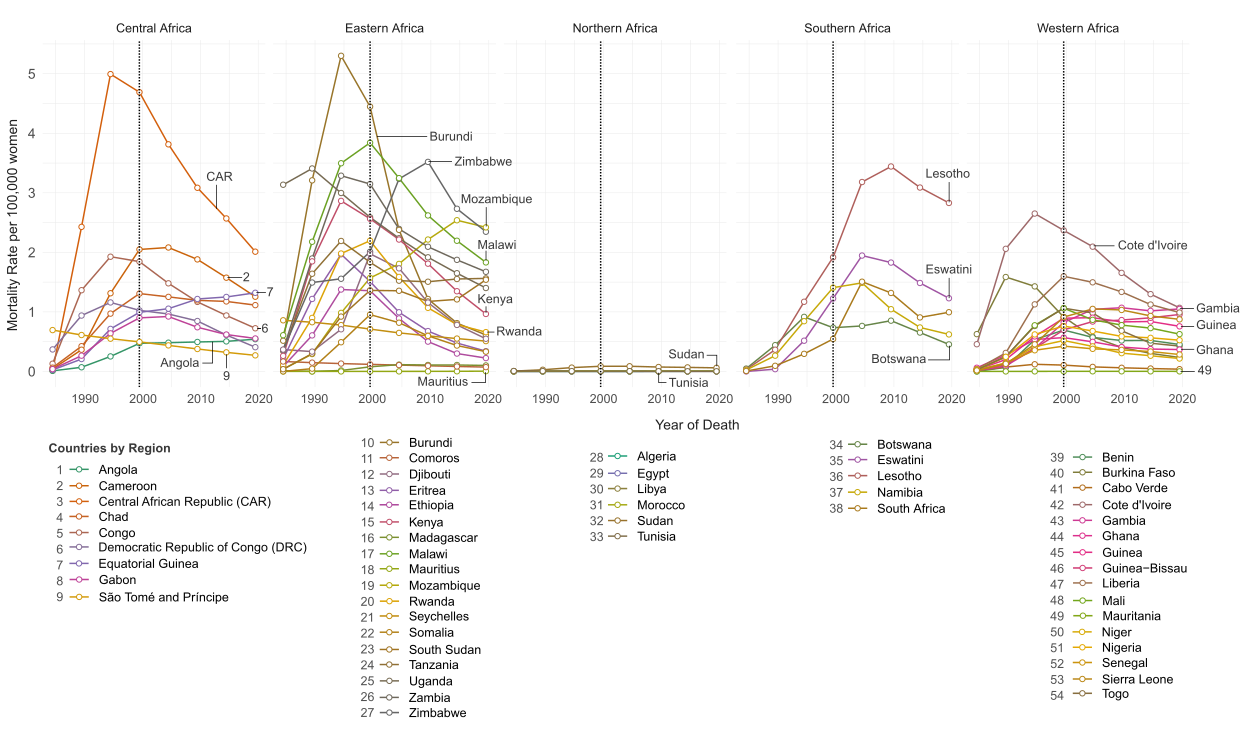
**

**Figure S1: Fitted temporal trends of HIV/AIDS‑related maternal mortality in the 30–34-year-old population This figure shows the model-adjusted expected rates of HIV/AIDS‑aggravated maternal mortality for women aged 30–34 years, calculated for each mid-year period from 1984.5 to 2019.5. The x-axis corresponds to calendar periods, and the y-axis represents the predicted rates. Different colours denote trends for the individual countries. The graph is anchored by the Abuja Summit reference period (1999.5), allowing for a clear comparison of changes over time.**
