# Supplementary material for: Two Decades and Counting Since the Abuja Summit: Where Do We Stand in the Fight Against HIV/AIDS-Related Maternal Mortality?
Source: Womens Health Rep (New Rochelle). 2025 Oct 8;6(1):1092–108. doi: 10.1177/26884844251386289 (PMC12549178; doi:10.1177/26884844251386289)
Supplement: Supplementary Figure S2 [file 26884844251386289_supplementary_figure_s2.docx]

**
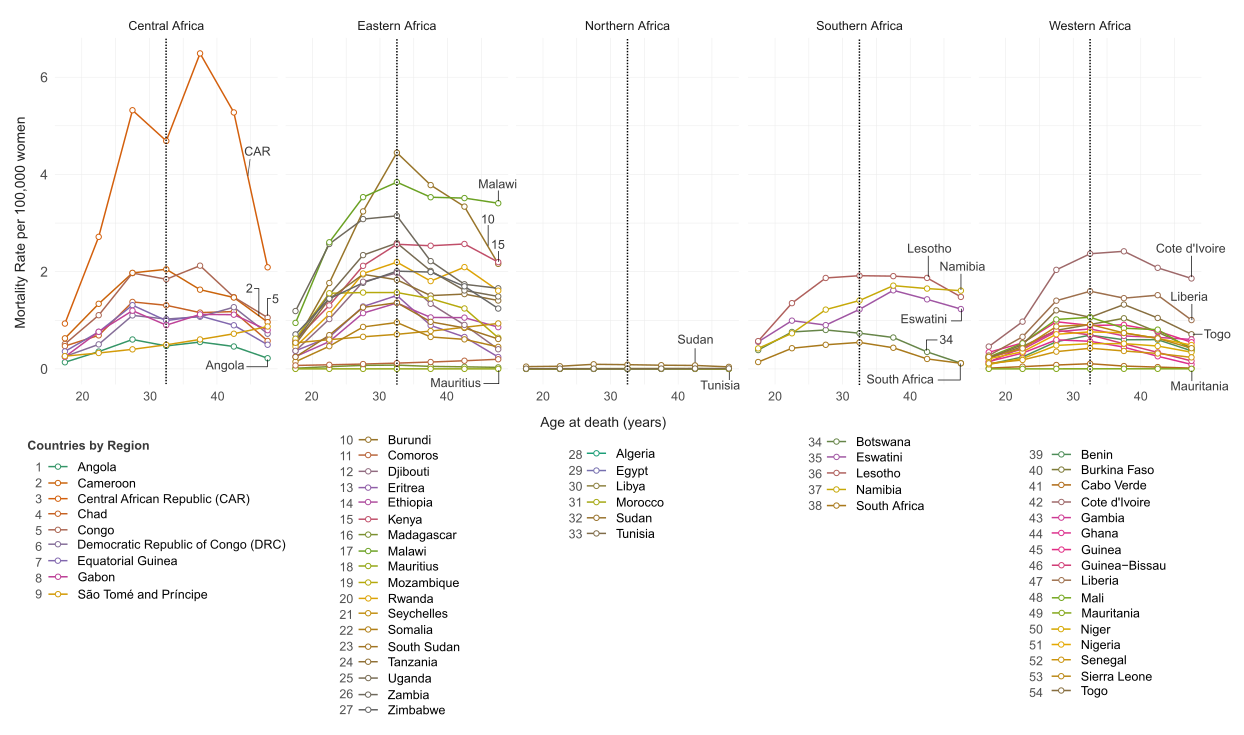
**

**Figure S2: Expected age‐specific HIV/AIDS‑aggravated maternal mortality rate (MMR) across a defined age range, adjusted for cohort effects. The horizontal axis (x‑axis) represents the age groups, while the vertical axis (y‑axis) displays the expected MMR values. Each point on the curve corresponds to the estimated MMR for women at that specific age, and the connecting line highlights the overall age‐related trend. Different colours distinguish curves for various countries, highlighting variations in the burden of MMR across diverse settings in Africa. The vertical dotted lines point to the central age group, 30-35 years, defined as the reference in all our age-period-cohort (APC) modelling.**
