# Supplementary material for: Two Decades and Counting Since the Abuja Summit: Where Do We Stand in the Fight Against HIV/AIDS-Related Maternal Mortality?
Source: Womens Health Rep (New Rochelle). 2025 Oct 8;6(1):1092–108. doi: 10.1177/26884844251386289 (PMC12549178; doi:10.1177/26884844251386289)
Supplement: Supplementary Figure S3 [file 26884844251386289_supplementary_figure_s3.docx]

**
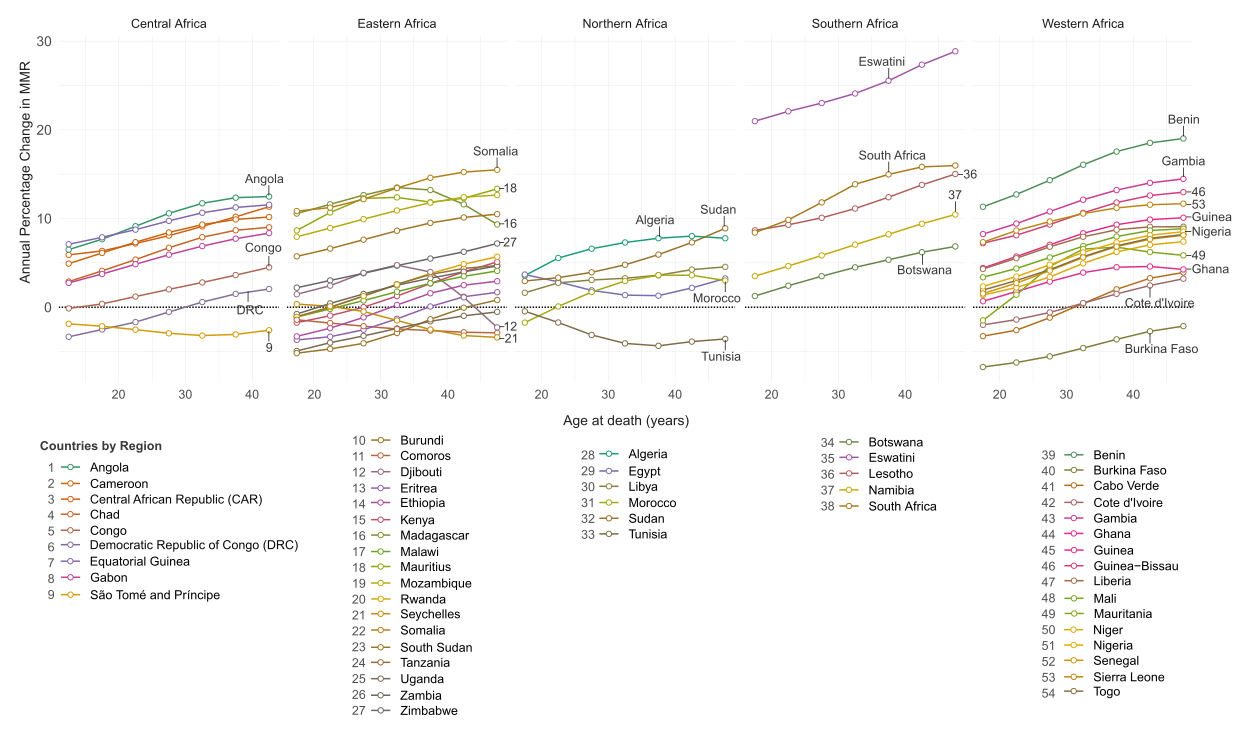
**

**Figure S3: Annual percentage change (APC) of the expected age-specific HIV/AIDS-aggravated maternal mortality rate (MMR) over time. The horizontal axis (x‑axis) represents the age groups, while the vertical axis (y‑axis) shows the APC values. Each point on the line corresponds to the estimated APC for women at that specific age, and the connecting line indicate the overall age‐related trend. A horizontal dotted line is drawn at the zero level on the y‑axis: values below zero indicate a declining trend in maternal mortality, independently from the overall trend, whereas values above signify an increasing trend. Different colours distinguish trends for various countries, highlighting variations in the trajectory and changes in MMR for the different age cohorts across Africa.**

**SS**
