# Supplementary material for: Two Decades and Counting Since the Abuja Summit: Where Do We Stand in the Fight Against HIV/AIDS-Related Maternal Mortality?
Source: Womens Health Rep (New Rochelle). 2025 Oct 8;6(1):1092–108. doi: 10.1177/26884844251386289 (PMC12549178; doi:10.1177/26884844251386289)
Supplement: Supplementary Figure S4 [file 26884844251386289_supplementary_figure_s4.docx]

**
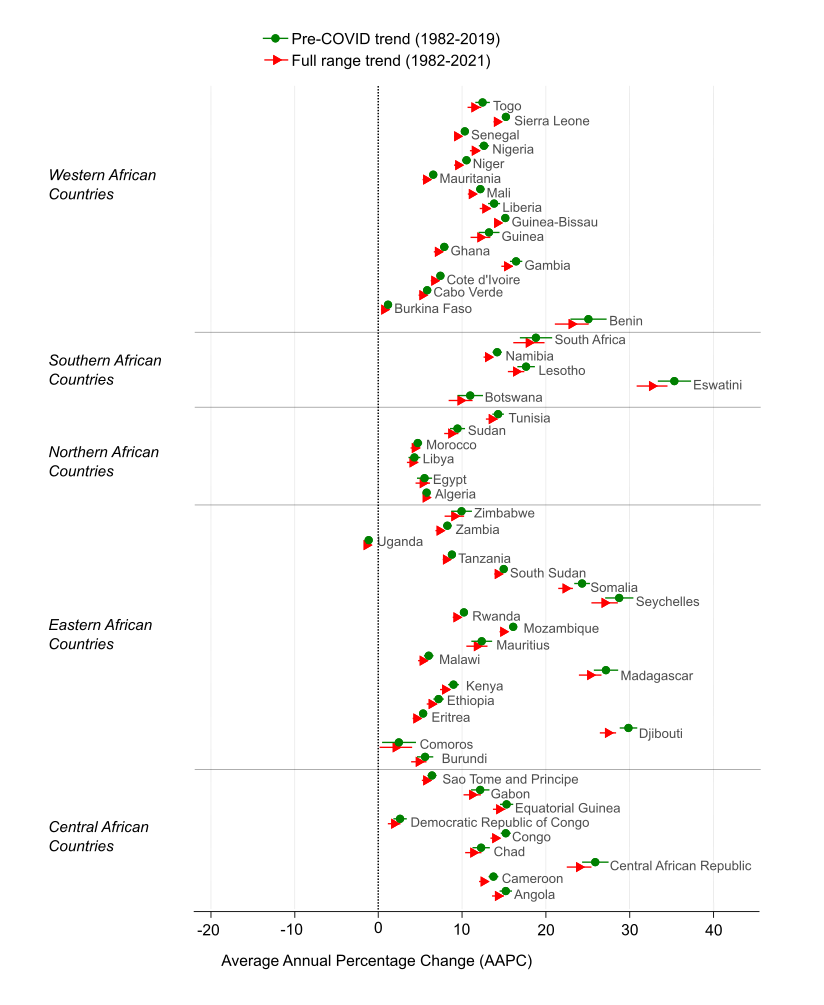
**

**Figure S4: This figure illustrates the temporal trends in HIV/AIDS-related maternal mortality across 54 African countries from 1982 to 2021, estimated using Joinpoint regression. The average annual percentage change (AAPC) quantifies the rate of change in the outcome (mortality), with positive values indicating an increasing trend and negative values showing a decline. Error bars represent 95% confidence intervals. Two estimates are presented: one for 1982–2019 (excluding COVID-19 pandemic era) and another for 1982–2021 (including the pandemic years – 2020 and 2021), to assess whether COVID-19 may have influenced the trends. The figure is stratified by geographic regions: Central, Eastern, Northern, Southern, and Western Africa. While some numerical shifts are observed, the overall direction remains stable, suggesting that COVID-19 did not significantly alter the trajectory, although longer-term data may be needed to fully capture potential delayed effects.**
